# Correction Algorithm for the Navigation System of an Autonomous Unmanned Underwater Vehicle

**DOI:** 10.3390/s20082365

**Published:** 2020-04-21

**Authors:** Danhe Chen, K. A. Neusypin, M. S. Selezneva

**Affiliations:** 1 Nanjing University of Science and Technology, Nanjing 210094, China; 2 Moscow Bauman State Technical University, Moscow 105005, Russia; neysipin@mail.ru (K.A.N.); m.s.selezneva@mail.ru (M.S.S.)

**Keywords:** unmanned underwater vehicle, inertial navigation system, measuring complex, nonlinear Kalman filter, SDC method, semi-natural experiment

## Abstract

More accurate navigation systems are always required for autonomous unmanned underwater vehicles (AUUV)s under various circumstances. In this paper, a measuring complex of a heavy unmanned underwater vehicle (UUV) was investigated. The measuring complex consists of an inertial navigation platform system, a Doppler lag (DL) and an estimation algorithm. During a relatively long-term voyage of an UUV without surfacing and correction from buoys and stationary stations, errors of the measuring complex will increase over time. The increase in errors is caused by an increase in the deviation angles of the gyro platform relative to the accompanying trihedron of the selected coordinate system. To reduce these angles, correction is used in the structure of the inertial navigation system (INS) using a linear regulator. To increase accuracy, it is proposed to take into account the nonlinear features of INS errors; an adaptive nonlinear Kalman filter and a nonlinear controller were used in the correction scheme. Considering that, a modified nonlinear Kalman filter and a regulator in the measuring complex are proposed to improve the accuracy of the measurement information, and modification of the nonlinear Kalman filter was performed through a genetic algorithm, in which the regulator was developed by the State Dependent Coefficient (SDC) method of the formulated model. Modeling combined with a semi-natural experiment with a real inertial navigation system for the UUV demonstrated the efficiency and effectiveness of the proposed algorithms.

## 1. Introduction

For performing underwater work as scientific research and practical implementation, unmanned underwater vehicles (UUV)s are widely used. The most famous UUV series are as follows: “Remus” (Hydroid, USA), “Autosub 6000” (UK), and “GAVIA” (Russia) [1,2]. With the help of UUVs, search operations and the inspection of sunk objects and pipelines are carried out, information warfare by creating false targets and interference is conducted, and UUVs are implemented for environmental monitoring, etc. Normally, UUVs can be classified according to the mass of the vehicle: micro, small, medium and heavy classes [3,4]. For the study of ice conditions, the performance of hydrographic work in the Arctic, as well as the fulfillment of special tasks in the interests of defense agencies, heavy autonomous unmanned underwater vehicles (AUUV)s (not towed UUVs) are applied. During the operation of UUVs, their exact orientation in space and knowledge of navigation parameters are very important. For this purpose, inertial navigation systems (INS), sonars, Doppler lags, etc., are installed on AUUVs [5,6,7]. For example, the UUV GAVIA is equipped with a strapdown INS and the WN-300 Doppler lag, which are combined into a measuring complex (MC). The INS and lag signals are processed together using the Kalman filter [8,9,10,11]. 

When the UUV is working under ice fields in the Arctic, there is no possibility of periodic ascent to the surface of the sea; thus, INS correction from a gyro-stabilized platform (GSP) is not provided. In the case of long-term autonomous navigation with the use of a strapdown INS, errors increase over time due to the instability of sensitive elements. Moreover, when UUVs perform maneuvers to complete tasks with long-term autonomous navigation, even for platform INS, errors will reach large values. This is due to an increase in the deviation angles of the gyro-stabilized platform (GSP) relative to the accompanying coordinate system (SC). Even with the correction of INS from a lag and information processing by the Kalman filter, errors of the navigation information increase as the model of INS errors in the Kalman filter becomes inadequate for the real process. 

Considering the prospective applications, scientists have been interested in AUUVs and all the particular constraints in different media have been formulated into mathematical problems. Wu et al. [12] generated the optimal paths based on the Particle Swarm Optimization (PSO) algorithm and the Kalman filter to finish an underwater target strike mission; Batista et al. [13] proposed a filtering method with applications to estimate the linear motion of underwater vehicles, taking into considertion both environmental disturbances and realistic measurement noise; Jens et al. proposed a sensor-based method with hybrid dynamical systems for underwater navigation; further, the observer performance should be evaluated in closed-loop with a feedback controller, an attitude observer, and a guidance scheme [14]. In order to improve the accuracy of navigation definitions of heavy UUVs for long-term autonomous operation, it is advisable to use more accurate nonlinear error models of INS in the algorithmic support. Accordingly, the nonlinear Kalman filter (NKF) can be applied in the MC [15,16]. 

In this work, the novelty lies in the application of algorithmic correction in the structure of the INS. Correction algorithms are proposed: an adaptive nonlinear Kalman filter and a nonlinear state regulator. The reduced regulator for INS correction in a nonlinear statement of the problem has been developed, and the Dependent Coefficient (SDC) method of transformation of the nonlinear INS error model is considered for the synthesis of the regulator; a correction in the structure of INS is proposed and used with the help of a regulator [17,18]. A reduced regulator and an adaptive regulator for the INS correction using linear models of INS errors were formulated, and the linear models of INS errors quite roughly describe the process of real INS errors. Moreover, the efficiency of the proposed INS correction method was demonstrated by mathematical modeling and simulation according to the semi-natural experiment.

The paper is organized as follows: In Section 2, the nonlinear errors model of INS for UUVs is introduced, as well as the platform of INS. The correction of navigation systems by a nonlinear Kalman filter modification is presented in Section 3, which provides the algorithmic support for the INS correction process of UUVs. Simulation and validation through experiments of the proposed method are performed in Section 4, and the discussion and analysis of results are presented later. Section 6 concludes the paper.

## 2. Nonlinear Errors Model of INS

The main measurement complex system of the studied UUV is INS; the platform INS consists of accelerometers installed on a GSP [19,20]. INS has errors which are caused by the drift of gyroscopes, accelerometers and other perturbing factors, and INS errors increase over time and could get large values. The platform coordinate system (CS) differs from the navigation CS in the misalignment angles $\phi_{E}$, $\phi_{N}$ and $\phi_{up}$. These angles can be used as indicators of system errors, considering the fact that the physical axes of the platform must coincide with the platform axes. Thus, the transition matrix from the navigation CS to the platform can be represented as [21,22,23,24]

(1) $$C_{LL}^{PL}=\left[\begin{array}{ccc} \cos\phi_{up} & \sin\phi_{up} & -\phi_{N}\\ -\sin\phi_{up} & \cos\phi_{up} & \phi_{E}\\ \phi_{N}\cdot \cos\phi_{up}+\phi_{E}\cdot \sin\phi_{up} & \phi_{N}\cdot \sin\phi_{up}-\phi_{E}\cdot \cos\phi_{up} & 1 \end{array}\right]$$

where PL denotes the platform trihedron; *LL* denotes the geographic trihedron.

The absolute angular velocity in the platform CS can be formulated using the angular velocity in the navigation CS and the derivatives of the mismatched angles.

(2) $${\left[\begin{array}{c} \omega_{E}\\ \omega_{N}\\ \omega_{up} \end{array}\right]}_{PL}=C_{LL}^{PL}{\left[\begin{array}{c} \omega_{E}\\ \omega_{N}\\ \omega_{up} \end{array}\right]}_{LL}+\left[\begin{array}{c} {\dot{\phi }}_{E}\\ {\dot{\phi }}_{N}\\ {\dot{\phi }}_{up} \end{array}\right]$$

The errors of INS horizontal accelerometers are obtained from the equations as follows:

(3) $${\left[\begin{array}{c} a_{E}\\ a_{N}\\ a_{up} \end{array}\right]}_{PL}=C_{LL}^{PL}{\left[\begin{array}{c} a_{E}\\ a_{N}\\ a_{up} \end{array}\right]}_{LL}+\left[\begin{array}{c} B_{E}\\ B_{N}\\ B_{up} \end{array}\right]+\left[\begin{array}{c} a_{E}\cdot \mu_{E}\\ a_{N}\cdot \mu_{N}\\ a_{up}\cdot \mu_{up} \end{array}\right]$$

where $B_{i}$ is zero offsets of accelerometers and $\mu_{i}$ is errors from scale coefficient.

Then, substituting Equation (1) into Equation (3), the error equations of INS can be obtained [21,22]:

(4) $$X_{k}=\Phi \cdot X_{k-1}+W_{k-1}+D$$

where $X_{k-1}=\left[\begin{array}{c} \delta V_{E}\\ \delta V_{N}\\ \phi_{E}\\ \phi_{N}\\ \phi_{up}\\ \delta \varphi \\ \delta \lambda \end{array}\right]$; $W_{k-1}=\left[\begin{array}{c} B_{E}+a_{E}\cdot \mu_{E}\\ B_{N}+a_{N}\cdot \mu_{N}\\ \omega_{E}^{dr}\\ \omega_{N}^{dr}\\ \omega_{up}^{dr}\\ 0\\ 0 \end{array}\right]$;

$$\Phi =\left[\begin{array}{ccccccc} \frac{1}{R}tg\varphi V_{N} & \left(2\cdot U\cdot \sin\varphi +\frac{V_{E}}{R}tg\varphi \right) & 0 & -a_{up} & a_{N} & \left(V_{N}\cdot U\cdot \cos\varphi +\frac{V_{N}^{2}}{R}\sec^{2}\varphi \right) & 0\\ -\left(2\cdot U\cdot \sin\varphi +\frac{V_{E}}{R}tg\varphi \right) & 0 & a_{up} & 0 & -a_{E} & -\left(\frac{V_{E}^{2}}{R}\sec^{2}\varphi +2\cdot V_{E}\cdot U\cdot \cos\varphi \right) & 0\\ 0 & -\frac{1}{R} & 0 & \omega_{up} & -\omega_{N} & 0 & 0\\ \frac{1}{R} & 0 & -\omega_{up} & 0 & \omega_{E} & -U\cdot \sin\varphi & 0\\ \frac{1}{R}tg\varphi & 0 & \omega_{N} & -\omega_{E} & 0 & \left(U\cdot \cos\varphi +\frac{V_{E}}{R}\sec^{2}\varphi \right) & 0\\ 0 & \frac{1}{R} & 0 & 0 & 0 & 0 & 0\\ \frac{1}{R\cdot \cos\varphi } & 0 & 0 & 0 & 0 & \frac{V_{E}}{R\cdot \cos\varphi }tg\varphi & 0 \end{array}\right]$$

$$D=\left[\begin{array}{c} -a_{E}\left(\frac{\phi_{up}^{2}}{2}-\frac{\phi_{up}^{4}}{24}\right)-a_{N}\left(\frac{\phi_{up}^{3}}{6}-\frac{\phi_{up}^{5}}{120}\right)\\ -a_{N}\left(\frac{\phi_{up}^{2}}{2}-\frac{\phi_{up}^{4}}{24}\right)+a_{E}\left(\frac{\phi_{up}^{3}}{6}-\frac{\phi_{up}^{5}}{120}\right)\\ \omega_{E}\left(\frac{\phi_{up}^{2}}{2}-\frac{\phi_{up}^{4}}{24}\right)+\omega_{N}\left(\frac{\phi_{up}^{3}}{6}-\frac{\phi_{up}^{5}}{120}\right)\\ \omega_{N}\left(\frac{\phi_{up}^{2}}{2}-\frac{\phi_{up}^{4}}{24}\right)-\omega_{E}\left(\frac{\phi_{up}^{3}}{6}-\frac{\phi_{up}^{5}}{120}\right)\\ \left(\omega_{E}\cdot \phi_{N}-\omega_{N}\cdot \phi_{E}\right)\left(\frac{\phi_{up}^{2}}{2}-\frac{\phi_{up}^{4}}{24}\right)-\left(\omega_{E}\cdot \phi_{E}-\omega_{N}\cdot \phi_{N}\right)\left(\phi_{up}^{}-\frac{\phi_{up}^{3}}{6}+\frac{\phi_{up}^{5}}{120}\right)\\ 0\\ 0 \end{array}\right]$$

where $V_{E}$, $\delta V_{E}$, $\delta V_{N}$ are velocities of the UUV in navigation CS and their errors; φ and δφ represent latitude and its error, respectively, *U* is the angular velocity of the Earth, and here, *D* is the nonlinear part.

In Equation (4), the matrix Φ determines the relation between the components of the state vector *x*. *W* is input noise, including dominant external disturbances. *D* is a matrix including nonlinear terms of the second order of smallness [21,22]. The dominant elements that determine the dynamics of *x* are selected in matrix Φ.

In the algorithmic support of measuring systems, which is implemented on the board of the underwater vehicle, more simple error models of INS are used [21,24]. For the only horizontal channel, the nonlinear error model of INS has its form as follows:

(5) $$x_{k}=Fx_{k-1}+w_{k-1}$$

where $x_{k}$ is the state vector, F is the matrix of the object and $w_{k}$ is input noise.

$$x_{k}={\left[\begin{array}{l} \delta v_{E}\\ \Phi_{N}\\ \omega_{N}^{dr} \end{array}\right]}_{k};F=\left[\begin{array}{ccc} 1 & -Tgcos\Phi_{E} & 0\\ \frac{T}{R} & 1-\frac{T}{R}\delta v_{N}tg\Phi_{E} & T\\ 0 & 0 & 1-T\beta \end{array}\right];w_{k}=\left[\begin{array}{l} TB_{E}\\ 0\\ TA\sqrt{2\beta }w \end{array}\right]$$

where $\delta v_{E}$ denotes error in determining the velocity, $\Phi_{NE}$ represents angles of horizontal deviation, $\omega_{N}^{dr}$ is drift velocity, $B_{E}$ is zero offset, and w is white noise.

In order to compensate for the INS errors, these errors should first be evaluated. Estimation of errors of INS is carried out using estimation algorithms [25], and one of the most common methods is the Kalman filter. Taking nonlinear components into account, the nonlinear Kalman filter (NKF) is used to estimate the INS errors. However, the INS error model in the NKF may not be adequate for the actual process of the changing the INS errors, especially when performing UUV maneuvers. Thus, the identification of the INS error model in the NKF should be considered.

## 3. Correction of Navigation Systems by a Nonlinear Kalman Filter Modification

When maneuvers are performed on UUVs, the deviation angles of GSP in INS relative to the selected coordinate system increase, and the linear model of its errors, obtained by taking into account the assumption of horizontal movement of the supporting object and small angles of stabilization, will become inadequate for the real process.

High-precision correction of navigation information is carried out by NKF and its modifications.

### 3.1. Methods of Realization of Nonlinear Kalman Filter

Assuming the equation for state vector has the next form:

(6) $$x_{k}=\Phi_{k}\left(x_{k-1}\right)+w_{k}$$

where $x_{k}$ represents the sate vector, $\Phi_{k}(x_{k-1})$ is the nonlinear model vector characterizing the dynamics of the process under study. Part of the state vectors are measured by INS and GPS navigation systems:

(7) $$z_{k}=H_{k}x_{k}+v_{k}$$

where $z_{k}$ is the measurement vector, $H_{k}$ is the matrix of measurement, and $w_{k}$ and $v_{k}$ are discrete analogs of Gaussian white noise with zero mathematical expectations and covariance matrices $Q_{k}$ and $R_{k}$, respectively, which are uncorrelated with each other.

The equations of the nonlinear Kalman filter have the following form [26,27]:

(8) $${\hat{x}}_{k}={\hat{x}}_{k,k-1}+K_{k}\left({\hat{x}}_{k-1}\right)\left[z_{k}-H_{k}{\hat{x}}_{k,k-1}\right]$$

(9) $${\hat{x}}_{k,k-1}=\Phi_{k}\left({\hat{x}}_{k-1}\right)$$

(10) $$K_{k}\left({\hat{x}}_{k-1}\right)=P_{k,k-1}H_{k}^{T}{\left[H_{k}P_{k,k-1}H_{k}^{T}+R_{k}\right]}^{-1})$$

(11) $$P_{k,k-1}=\frac{\partial \Phi_{k}\left({\hat{x}}_{k-1}\right)}{\partial x_{k-1}^{T}}P_{k-1}{\left[\frac{\partial \Phi_{k}\left({\hat{x}}_{k-1}\right)}{\partial x_{k-1}^{T}}\right]}^{T}+Q_{k}$$

(12) $$P_{k}=\left[I-K_{k}\left({\hat{x}}_{k-1}\right)H_{k}\right]P_{k,k-1}$$

where $H_{k}\left({\hat{x}}_{k-1}\right)$ is the matrix of gain coefficients of the Kalman filter. 

Such an approach can be applied only in the case of a unimodal character of a posteriori density. When the posterior density is multi-extreme, an algorithm can be used where the posterior density is represented by a set of δ functions.

The listed implementations of the Kalman nonlinear filter require linearization of the INS error model using the Taylor series, representing the posterior density as a set of δ functions, or replacing the posterior density with a system of partial Gaussian densities with different weights. As a result, only linear models of INS errors are used in the Kalman filter. The nonlinear Kalman filter models applied in the general case are difficult due to the fact that the posterior density of the state vector is not Gaussian. Consequently, it is not possible to obtain algorithmic recurrence relations for calculating estimations of the state vector.

The other famous methods for the implementation of the Kalman filter is solving a stochastic partial differential equation written in the Ito or Stratonovich form. However, the practical implementation of this solution is also complicated; special rules which do not coincide with the usual rules of mathematical analysis need to be applied when integrating these equations. Another disadvantage of the approach of the non-linear Kalman filter mentioned is that it has lower accuracy than the original non-linear model. The most complete account of all the nature features of changes in INS errors, and most importantly, a specific INS in conditions of each specific flight, it is possible by constructing a nonlinear model using one of the evolutionary algorithms.

Therefore, the nonlinear model can be used as a reference model to ensure the adequacy of the Kalman filter model and the real change process of INS errors. Figure 1 presents a scheme of INS correction by genetic algorithm (GA) [28,29], where NKF denotes the nonlinear Kalman filter, and C denotes the divergence indicator of the evaluation process. 

For a neural network in the Kalman filter, we can apply an indicator to the network, which is considered as the sum of squared residuals for all components of the output vector and all sets of measurements between the reference values and values at the output of the neural network. Meanwhile, the RAO-Kramer inequality can be used as an indicator of divergence in the INS correction scheme.

As a modification of Kalman filter, GA is applied to build a model of the evaluation process. If the evaluation becomes divergent, then a new model will be used in the Kalman filter. Thus, the developed correction algorithm in accordance with Fig. 1 consists of a non-linear Kalman filter, an estimation indicator, and GA, and we have a model of the process under study (INS error model) at the output side [26].

The following notations are introduced in Figure 1: NKF represents the non-linear Kalman filter; **θ** represents true navigation information; **ξ** is DL error vector; x is the vector of INS errors; z is the vector of measurements; $\hat{x}$ denotes the estimation of the error vector of the INS; $\tilde{x}$ denotes the estimation of INS errors.

Here, GA is implemented as a standard algorithm, information about INS error estimation is obtained by the modified NFC, and correction signals are generated in the structure of INS.

In accordance with Figure 1, INS correction is carried out in the output signal, and the correction signal does not affect the dynamics of INS. Deviation angles of GSP increase and the model of INS errors becomes inadequate for the real process over time. Therefore, it is necessary to reduce the angles of deviation of GSP and maintain the adequacy of the INS error model, considering that corrective signals are forwarded into the INS structure. When the information about estimation of INS errors is obtained by the modified NKF, correction signals will be generated in the structure of INS.

### 3.2. Correction in the Structure of INS

The implementation of INS involves obtaining not only the navigation parameters of the object, but also information about its orientation relative to the reference coordinate system. The reference coordinate system is defined by the GSP. However, the GSP deviates from the given position due to the drift of gyroscopes, zero offset, accelerometer drift, and errors of the first integrator. A significant increase in the deflection angles of the GSP leads to platform drift due to moments of residual imbalance around the precession axes of the gyroscopes and the anisoelasticity of the GSP and gyroscopes during vibration. As errors of autonomous INS increase over time, in order to obtain reliable information about the orientation of the NPA, it is necessary to compensate for the deviation of the GSP from the specified position.

The correction scheme in the structure of INS is presented in Figure 2:

### 3.3. Development of a Nonlinear Algorithm for INS Errors Correction

The INS error equation can be described in Equation (5), and SDC representation of a nonlinear system (5) has the following form:

(13) $$x_{k}=Fx_{k}+w_{k}$$

where $x_{k}=\left[\begin{array}{c} \delta V_{k}\\ \psi_{k}\\ \varepsilon_{k} \end{array}\right]$; $w_{k}=\left[\begin{array}{c} B_{k}\\ 0\\ \eta_{k} \end{array}\right]$; $F=\left[\begin{array}{ccc} 1 & -Tg_{k} & 0\\ \frac{T}{R_{k}} & 1+\frac{T\delta V_{k}}{R_{k}} & T\\ 0 & 0 & 1-T\mu \end{array}\right]$ and *T* is the period of discretization.

The state vector is represented as the sum of vectors $z_{k}$ and $y_{k}$, which are extracted in vector $z_{k}$,the only components that we intend to control, and in vector $y_{k}$, all remaining components of the state vector. The equation of the object has the following form:

(14) $$x_{k}=Fz_{k-1}+Gy_{k-1}+w_{k-1}+u_{k-1}$$

and denotes:

(15) $$w_{k-1}+Gy_{k-1}=\zeta_{k-1}$$

Assuming that $z_{k-1}$ and $\zeta_{k-1}$ can be evaluated, the control will be searched in the form as follows:

(16) $$u_{k-1}=-\left(K_{k-1}{\hat{z}}_{k-1}+{\hat{\zeta }}_{k-1}\right)$$

The implementation of state vector estimation in the regulator implies a preliminary assessment by using the estimation algorithm. At the output of the estimation algorithm, we have a signal as follows:

(17) $${\hat{x}}_{k}=x_{k}-{\tilde{x}}_{k}$$

where ${\tilde{x}}_{k}$ represents error estimation of the state vector.

Substituting Equation (16) into Equation (14) and taking into account expression (17), we can obtain:

(18) $$x_{k}=\left(F-K_{k-1}\right)z_{k-1}+K_{k-1}{\tilde{z}}_{k-1}+{\tilde{\zeta }}_{k-1}$$

The optimal control is determined by finding a regulator matrix in which the functional is

(19) $$J=M\left[x_{k}{}^{T}x_{k}\right]$$

Here we will take the minimum value of this function. The covariance matrix of state vector can be represented as follows:

(20) $$M\left[x_{k}x_{k}{}^{T}\right]=M\left\{\left[\left(F-K_{k-1}\right)x_{k-1}+K_{k-1}{\tilde{x}}_{k-1}+{\tilde{\zeta }}_{k-1}\right]\times {\left[\left(F-K_{k-1}\right)x_{k-1}+K_{k-1}{\tilde{x}}_{k-1}+{\tilde{\zeta }}_{k-1}\right]}^{T}\right\}$$

Considering the principle of orthogonality, Equation (20) takes the form of

(21) $$\begin{array}{ll} M\left[x_{k}^{T}x_{k}\right] & =\left(F-K_{k-1}\right)M\left[x_{k-1}x_{k-1}^{T}\right]{\left(F-K_{k-1}\right)}^{T}+\left(F-K_{k-1}\right)M\left[{\tilde{x}}_{k-1}{\tilde{x}}_{k-1}^{T}\right]K_{k-1}^{T}+\\ & +K_{k-1}M\left[{\tilde{x}}_{k-1}{\tilde{x}}_{k-1}^{T}\right]{\left(F-K_{k-1}\right)}^{T}+K_{k-1}M\left[{\tilde{x}}_{k-1}{\tilde{x}}_{k-1}^{T}\right]K_{k-1}^{T}+\left(F-K_{k-1}\right)M\left[{\tilde{x}}_{k-1}{\tilde{\zeta }}_{k-1}^{T}\right]\\ & +M\left[{\tilde{\zeta }}_{k-1}{\tilde{x}}_{k-1}^{T}\right]{\left(F-K_{k-1}\right)}^{T}+K_{k-1}M\left[{\tilde{x}}_{k-1}{\tilde{\zeta }}_{k-1}^{T}\right]+M\left[{\tilde{\zeta }}_{k-1}{\tilde{x}}_{k-1}^{T}\right]K_{k-1}^{T}+\\ & +K_{k-1}M\left[{\tilde{x}}_{k-1}{\tilde{x}}_{k-1}^{T}\right]K_{k-1}^{T}+\left[{\tilde{\zeta }}_{k-1}{\tilde{\zeta }}_{k-1}^{T}\right] \end{array}$$

Let us determine the sum of variances of the state vector:

(22) $$J=sp\;M\left[x_{k}x_{k}{}^{T}\right]=M\left[x_{k}{}^{T}x_{k}\right]$$

Then, the optimal value of regulator matrix on the condition of zero gradient can be obtained:

(23) $$\frac{\partial J}{\partial K_{k-1}}=0$$

Using the matrix differentiation rules, an optimality condition that leads to a minimum of the functional can be obtained:

(24) $$K_{k-1}=F$$

Thus, a MC with correction in the structure of INS is developed in this section. In order to improve the accuracy of navigation definitions of UUV, the nonlinear control algorithm and the developed control algorithm based on SDC representation of the nonlinear model of INS errors are applied in the MC.

## 4. Experimental Study and Validation

In order to conduct an experiment with a real INS, the following steps should be considered and performed:

1. Install the INS platform on a fixed base, and enable INS. Since the INS is stationary, the output signal is an INS error.
2. Generate measurements for the Kalman filter z in accordance with Figure 1, and a Doppler lag error is simulated using a random number sensor. The signal is smoothed at the interval *T* = 12 s.
3. Evaluate the INS errors by means of the algorithm in Figure 1. In the Kalman filter in the matrix F, the following numerical values are used: *R* = 6,370,000 m, *g* = 9.8 m/s^2^, the average frequency of random drift change is µ = 2 × 10^−4^/s. In Equation (10), during the formation of the covariance matrix of the Kalman filter measuring noise, errors of the Doppler lag v are assumed to be white noise with an intensity equal to 0.015 m/s, and in Equation (11) in the matrix Q, the zero offset of the accelerometer is assumed to be a constant value *B* = 5 × 10^−4^ m/s^2^; the dispersion of external perturbation on the gyroscope η*_k_* is assumed to be 10^−16^.
4. Form the control *u* = $-F\hat{x}$, and submit a signal *u* to the input of INS with Figure 2. The control signal is forwarded to the input of first integrator and the input of the torque sensor.

The results of the operation modeling of MC with a control algorithm and MC with a nonlinear control algorithm are presented in Figure 3, Figure 4, Figure 5 and Figure 6.

In Figure 3 and Figure 5, it is shown that the errors of the MC in INS with the linear and nonlinear models that were used in the regulator at the first stage almost coincide. The difference begins to be clearly observed with 30 min of MC operation. Based on the results of mathematical modeling, it can also be seen that it is possible to improve the accuracy of error calculation in determining velocity by 10%, and deviation angles of GSP by 15%.

For comparison and validation, modeling according to a laboratory experiment was carried out using a nonlinear Kalman filter. The simulation results are presented in Figure 7, Figure 8 and Figure 9.

Figure 6 and Figure 7 show the results of modeling the INS error in autonomous mode and with correction obtained by the control algorithm. Line 1 represents the error of INS in determining the speed in autonomous mode (without correction), and line 2 shows the INS error in determining the velocity after correction using the developed nonlinear control algorithm.

In Figure 8, line 1 denotes the deviation angles of a real system GSP and line 2 represents the estimation of deviation angles of the GSP.

In Figure 9, line 1 denotes the velocity drift of a real system GSP, and line 2 represents the estimation of deviation angles of the GSP.

## 5. Discussion and Analysis of Simulation Results

Here, a set of algorithms was developed for MC, including a nonlinear control algorithm that was used for error compensation in the structure of INS, and they can work for a long time without correction from stationary navigation stations. In the synthesis of the control algorithm, the method of SDC representation of nonlinear models was used. The efficiency and effectiveness of the developed algorithms were demonstrated by means of mathematical modeling and simulations according to data results of the semi-natural experiment within the TS060K system (Inertial Navigation System Ц060K, Russia).

The proposed MC correction algorithms increase the accuracy of navigational determinations of UUVs during long-term voyages in the underwater state and without correction from stationary stations and buoys. While the proposed algorithms have applied a priori models in the regulator, which may not accurately describe the changing process of INS errors, in general, the SDC method has limitations in its application [30,31], and not every model can be represented by the SDC method. These aspects limit the application of the developed algorithmic software in other dynamic objects.

## 6. Conclusions

In this paper, the measurement complex of an autonomous heavy UUV designed for long voyages without surfacing on the sea surface was developed. The MC consists of an INS platform, Doppler lag, NKF of modified GA, and a nonlinear controller for correction in the structure of INS. Synthesis of the reduced regulator was carried out using the SDC method. 

With the help of a regulator, INS errors were reduced during operation for a long time without correction from stationary navigation stations, GPS and beacons. In comparison with the well-known methods (linear optimal and adaptive regulators in the INS correction scheme), the simulation results confirm the advantages of the proposed method for increasing the accuracy of INS. Therefore, the method proposed in this paper allows an increase of the accuracy of navigational determinations of UUVs during long-term (more than 3 hours) autonomous navigation.

## Figures and Tables

**Figure 1:**
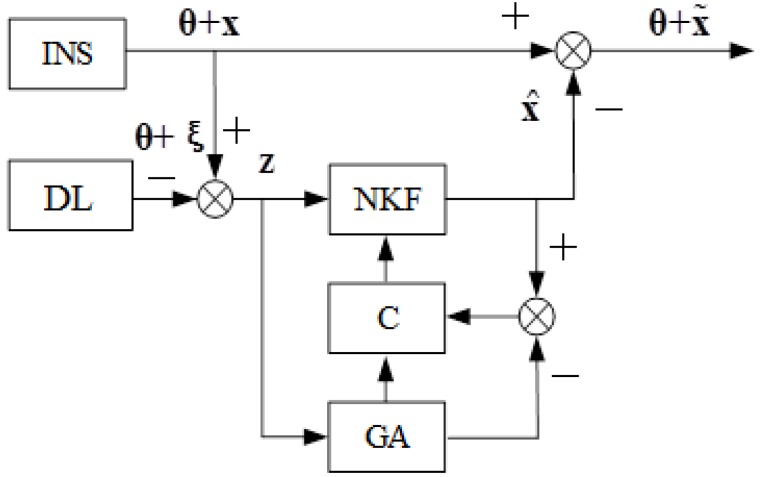
Scheme of inertial navigation system (INS) correction by genetic algorithm (GA).

**Figure 2:**
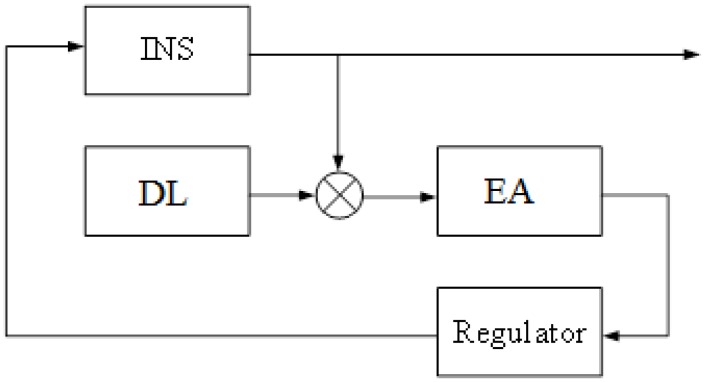
Scheme of connection to the INS estimation algorithm and regulator.

**Figure 3:**
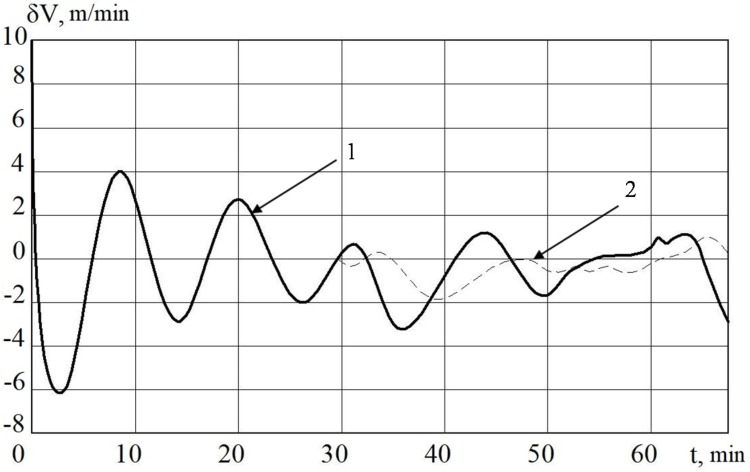
INS errors in determining the velocity with regulator (1) and non-linear regulator (2).

**Figure 4:**
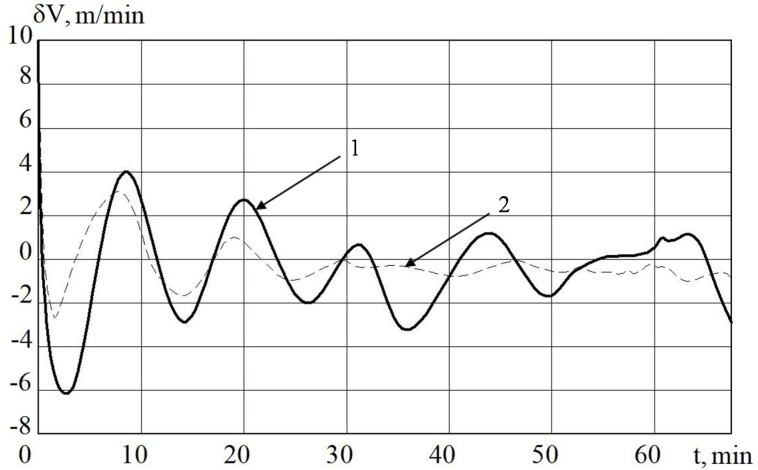
INS errors in determining the velocity MC with regulator (1) and non-linear SDC regulator (2).

**Figure 5:**
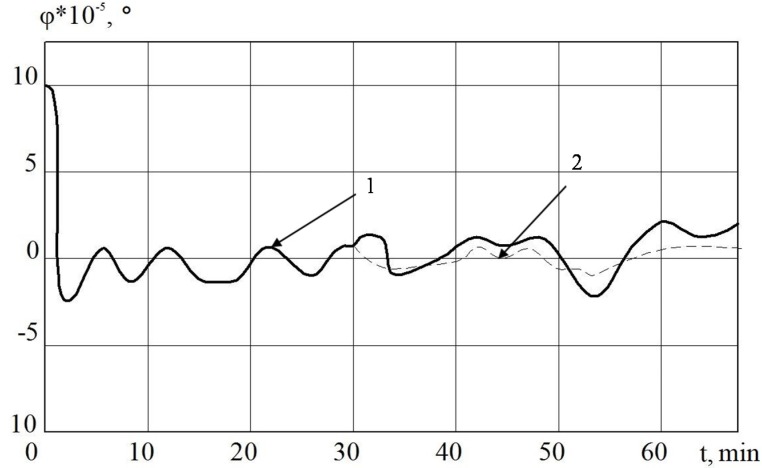
The deviation angle of the gyro-stabilized platform (GSP) in INS from the horizon plane when using MC with a regulator (1) and a non-linear regulator (2).

**Figure 6:**
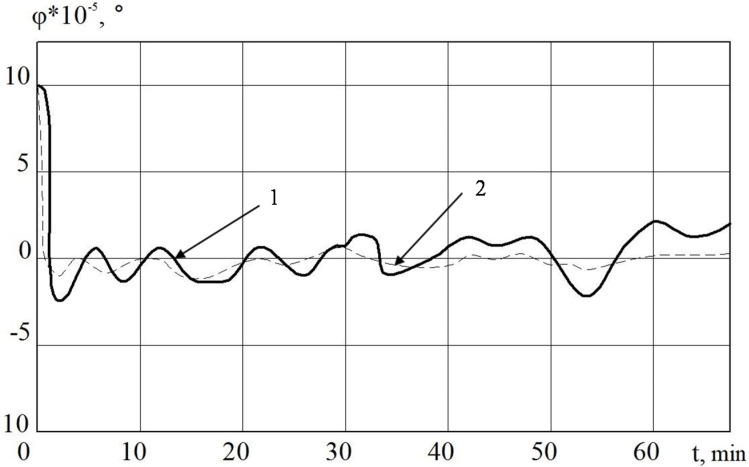
The deviation angle of the GSP in INS from the horizon plane when using a measuring complex (MC) with a regulator (1) and a non-linear SDC regulator (2).

**Figure 7:**
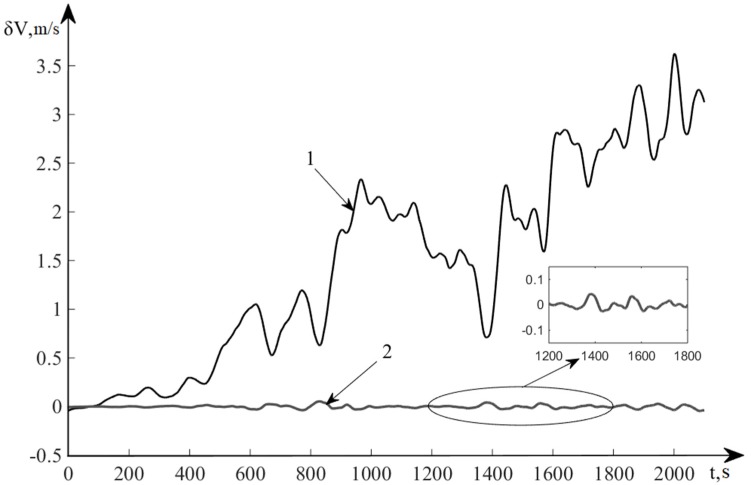
TS060K Errors for determination of velocity.

**Figure 8:**
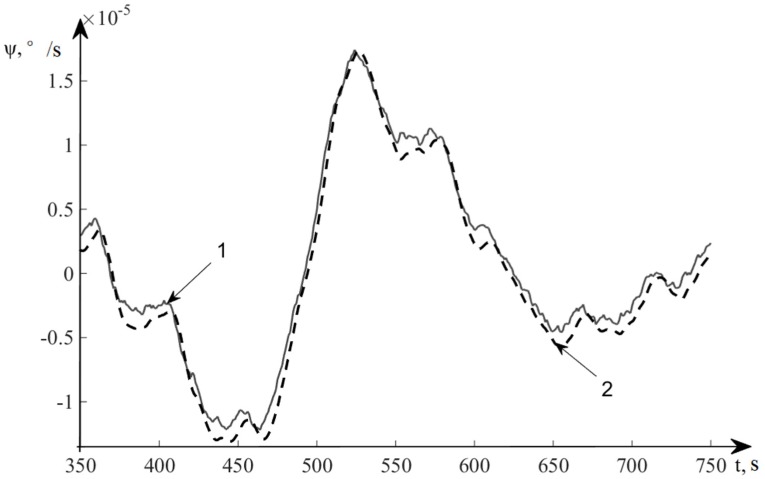
Estimation results of deviation angles of the GSP.

**Figure 9:**
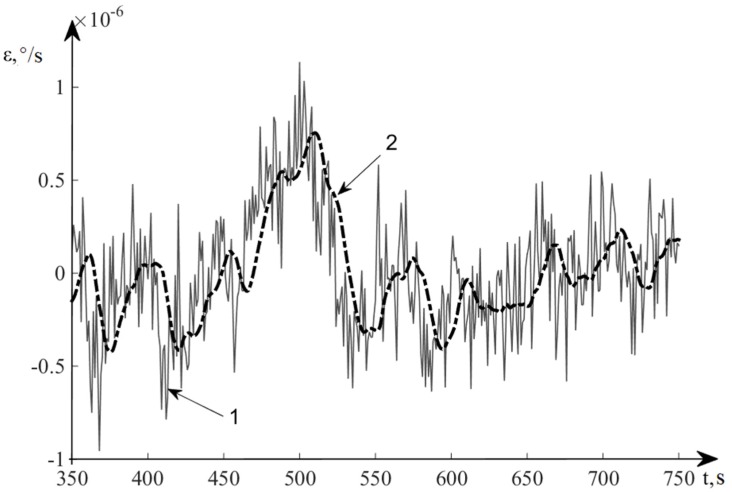
Estimation results of velocity drift of the GSP.
